# Machine Learning Models Derived from [^18^F]FDG PET/CT for the Prediction of Recurrence in Patients with Thymomas

**DOI:** 10.3390/bioengineering12070721

**Published:** 2025-06-30

**Authors:** Angelo Castello, Luigi Manco, Margherita Cattaneo, Riccardo Orlandi, Lorenzo Rosso, Giorgio Alberto Croci, Luigia Florimonte, Giovanni Scribano, Alessandro Turra, Stefano Ferrero, Mario Nosotti, Gianpaolo Carrafiello, Massimo Castellani, Paolo Mendogni

**Affiliations:** 1 Department of Nuclear Medicine, Fondazione IRCCS Ca’ Granda, Ospedale Maggiore Policlinico, 20122 Milan, Italy; angelo.castello@policlinico.mi.it (A.C.); luigia.florimonte@policlinico.mi.it (L.F.); massimo.castellani@policlinico.mi.it (M.C.); 2 Medical Physics Unit, University Hospital of Ferrara, 44124 Ferrara, Italy; luigi.manco@ausl.fe.it (L.M.); giovanni.scribano@edu.unife.it (G.S.); a.turra@ospfe.it (A.T.); 3 Division of Thoracic Surgery and Lung Transplantation, Fondazione IRCCS Ca’ Granda, Ospedale Maggiore Policlinico, 20122 Milan, Italy; margherita.cattaneo90@gmail.com (M.C.); riccardo.orlandi@unimi.it (R.O.); lorenzo.rosso@unimi.it (L.R.); mario.nosotti@unimi.it (M.N.); 4 Department of Pathophysiology and Transplantation, University of Milan, 20122 Milan, Italy; 5 Division of Pathology, Fondazione IRCCS Ca’ Granda, Ospedale Maggiore Policlinico, 20122 Milan, Italy; giorgio.croci@policlinico.mi.it (G.A.C.); stefano.ferrero@policlinico.mi.it (S.F.); 6 Department of Biomedical, Surgical and Dental Sciences, University of Milan, Via Festa del Perdono 7, 20122 Milan, Italy; 7 Department of Diagnostic and Interventional Radiology, Fondazione IRCCS Ca’ Granda, Ospedale Maggiore Policlinico, Via Francesco Sforza 35, 20122 Milan, Italy; gianpaolo.carrafiello@unimi.it; 8 Faculty of Health Science, Università degli Studi di Milano, Via Festa del Perdono 7, 20122 Milan, Italy

**Keywords:** thymomas, FDG, PET/CT, recurrence, radiomics, machine learning

## Abstract

**Background/Objectives:** This study aimed to develop machine learning (ML) models to predict recurrence in thymoma patients using conventional and radiomic signatures extracted from preoperative [^18^F]FDG PET/CT. **Methods:** A total of 50 patients (25 males, 25 females; mean age 63.3 ± 14.2 years) who underwent thymectomy and preoperative [^18^F]FDG PET/CT between 2012 and 2022 were retrospectively analyzed. Radiomic analysis was performed using free-from-recurrence (FFR) status as a reference. Clinico-metabolic PET parameters were collected, and thymoma lesions were manually segmented on [^18^F]FDG PET/CT. A total of 856 radiomic features (RFts) were extracted from PET and CT datasets following IBSI guidelines, and robust RFts were selected. The dataset was split into training (70%) and validation (30%) sets. Two ML models (PET- and CT-based, respectively), each with three classifiers—Random Forest (RF), Support-Vector-Machine, and Tree—were trained and internally validated using RFts and clinico-metabolic signatures. **Results:** A total of 50 ROIs were selected and segmented. FFR was observed in 84% of our cohort. Forty-three robust RFts were selected from the CT dataset and 16 from the PET dataset, predominantly wavelet-based RFts. Additionally, three metabolic PET parameters were selected and included in the PET Model. Both the CT and PET models successfully discriminated against FFR after surgery, with the CT Model slightly outperforming the PET Model across different classifiers. The performance metrics of the RF classifier for the CT and PET models were AUC = 0.970/0.949, CA = 0.880/0.840, Precision = 0.884/0.842, Recall = 0.880/0.846, Specificity = 0.887/0.839, Sensitivity = 0.920/0.844, TP = 81.8%/83.3%, and TN = 92.9%/84.6%, respectively. **Conclusions:** ML-models trained on PET/CT radiomic features show promising results for predicting recurrence in patients with thymomas, which could be potentially applied in clinical practice for a better personalized treatment strategy.

## 1. Introduction

Thymic epithelial tumors (TETs) are relatively rare tumors, although the most common of the anterior mediastinum, accounting for 0.15–0.32 cases per million in adults [1]. According to World Health Organization (WHO) classification thymomas, divided in five subtypes (i.e., A, AB, B1, B2, and B3), and thymic carcinomas are the main hystological forms [2]. In addition, the histotypes determine the prognosis, with types A, AB, or B1 (low-risk thymomas) characterized by longer survival than types B2, B3 (high-risk thymomas) and thymic carcinomas [3,4,5].

Therefore, an accurate pre-operative strategy is fundamental for patients’ risk stratification and prognostication in order to choose the appropriate treatment for TETs patients. Computed tomography (CT) is the standard imaging technique recommended by European Society of Molecular Oncology for the diagnosis of TET, although it often struggles to distinguish histologic subtypes due to overlapping morphological features [6]. In the last decade, several studies have been published on the role of fluorine-18-fluorodeoxyglucose ([^18^F]FDG) positron emission tomography (PET) for predicting aggressive histopathology and stage in TETs [7,8,9,10,11]. Notably, a recent meta-analysis showed that SUVmax was significantly higher in patients with high-risk thymomas (HRT) and thymic carcinomas than low-risk thymomas (LRT). Nevertheless, a clear cut-off value was not identified due to large overlap among TETs groups as for CT imaging [10]. In addition, more uncertainties exist regarding the use of volumetric-PET parameters, such as metabolic tumor volume (MTV) and total lesion glycolysis (TLG) to predict TETs histology and prognosis [12,13]. On the other hand, few studies have explored the role of [^18^F]FDG PET/CT for prognosis evaluation, mainly due to the paucity of cases of PET performed in patients with TETs, as well as the need for a long follow-up [14,15]. For this reason, the International Thymic Malignancy Interest Group (ITMIG) has proposed that freedom-from-recurrence (FFR) is a more meaningful metric than overall survival for patients who undergo curative-intent treatment. This recommendation is based on the slow-growing nature of thymomas, with approximately only 50% of deaths in these patients directly attributed to thymomas [16].

Radiomics is an emerging innovative technique, based on the extraction of numerous features from medical images through different mathematical models, able to provide biological information. Similarly, machine-learning (ML) and deep-learning (DL) have the potential to learn and to detect interactions among various data types in order to make predictions as accurate as possible [17,18,19]. However, to our knowledge, few studies has investigated the usefulness of a radiomics or ML approach, based on [^18^F]FDG images, in patients with TETs [20,21,22].

Therefore, our study aimed to examine whether the ML approach, using [^18^F]FDG PET/CT radiomic and DL features, is useful for predicting clinical outcomes of patients with TETs.

## 2. Materials and Methods

### 2.1. Study Population

Patients referred to our Hospital (Fondazione IRCCS Cà Granda Ospedale Maggiore Policlinico, Milan, Italy) were retrospectively analyzed. From May 2012 to November 2022, all participants with suspected thymic lesion underwent pre-operative [^18^F]FDG PET/CT scan. Clinical and surgical data were reviewed for each patient, including the presence or not of Miastenia Gravis, the modified Masaoka–Koga system stage, as well as the 8th TNM stage. All thymic tumors were classified based on the fifth edition of the WHO classification of Thoracic Tumors, as follows: LRT, HRT, and thymic carcinoma. Types A, AB, B1 were included in the LRT groups, while B2, B3 and thymic carcinomas in the HRT group [3].

The study was approved by the local Ethics Committee and institutional review board (Study identification: INT121/24). All patients were previously informed and signed their agreement before undergoing [^18^F]FDG PET/CT, in accordance with the Declaration of Helsinki.

### 2.2. Follow-Up

After surgery, all patients were followed up by clinical examination and imaging data, such as conventional imaging and/or a second [^18^F]FDG PET/CT scan, every 3 or 6 months according to tumor board’ indications, in order to determine the presence/absence of recurrence. As abovementioned, following ITMIG recommendations and due to indolent behavior of TETs, we used FFR as surrogate factor for overall survival in our cohort. Patients were dichotomized as FFR0 or FFR1 according to the absence or evidence of recurrence, respectively.

### 2.3. [^18^F]FDG PET/CT Imaging

[^18^F]FDG PET/CT was performed according to current European Association of Nuclear Medicine guidelines; the administered [^18^F]F-FDG activity was set according to patient’s Body Mass Index. Patients fasted for at least 6 h, and their blood glucose levels were <200 mg/dL before intravenous administration of [^18^F]F-FDG. Images were acquired after 60 ± 10min of uptake time using Biograph 64 Truepoint and Biograph Vision 450 PET/CT scanners (both Siemens Healthineers, Erlangen, Germany).

The [^18^F]FDG PET/CT images were critically analyzed by two experienced nuclear medicine physicians (A.C. and M.C.) on a syngo.via workstation (Siemens Healthineers, Enlargen, Germany). Pathological findings were considered to be area(s) of focal increased radiotracer uptake outside the sites of physiological distribution. Any discrepancies were resolved by consensus. Circular manual regions of interest (ROIs) were drawn around the pathological findings and automatically converted into 3D volumes of interest (VOIs) by the software. Semi-quantitative parameters were calculated as previously described, briefly [23]: SUVmax, SUVmean, SUVpeak of the tumor lesion, as well as volumetric parameters as Metabolic Tumor Volume (MTV) and Total Lesion Glycolysis (TLG). In addition, other semi-quantitative parameters were calculated to reduce the variability due to different PET/CT scanners, such as rPET (i.e., tumor SUVmax/LiverSUVmax); qPET (i.e., tumor SUVpeak/LiverSUVmean); and T/M, defined as the ratio between the SUVmax of thymomas and the SUVmean of aortic arc taken as reference for background activity.

### 2.4. Imaging Post-Processing and Features Extraction

**Figure 1:**
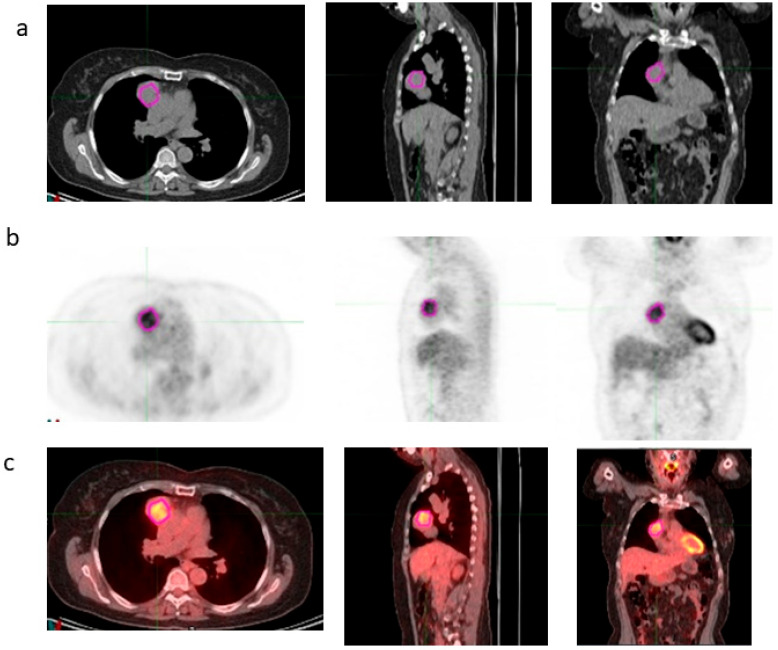
Manual segmentation of the VOI on transaxial CT (**a**), PET (**b**) and fused (**c**) images around the area of thymus using MiM Maestro software version 7.3.2.

VOI segmentations of the thymic lesions were manually performed on [^18^F]FDG PET/CT images by two expert nuclear medicine physicians on MIM maestro version 7.3.2 (MIM Software, Inc., Cleveland, OH, USA), as shown in Figure 1. Any discrepancies were resolved by consensus.

From each VOIs on PET and CT images, quantitative radiomics features (RFts) were extracted. The process was performed separately using the Radiomics package 3.12 and 3D Slicer image computing platform, according to IBSI standardization [24].

The fix bin width method was applied in both CT and PET images, the width of 25 was considered and for each VOI, 121 RFts were extracted from the original images. Among those, 10 RFts were excluded because they pertain to shape 2D or to python 3.12 package information. The RFts are divided by classes: 14 RFts belong to original image and mask, 14 to Shape (3D) class, 18 to First Order intensity statistics, 24 to Gray Level Co-occurrence Matrix (GLCM), 16 to Gray Level Run Length Matrix (GLRLM), 16 to Gray Level Size Zone (GLSZM), 14 to Gray Level Dependence Matrix (GLDM), and 5 to Neighbouring Gray Tone Difference Matrix (NGTDM). In addition, 744 textural RFts were extracted from wavelet decomposed VOIs. In total, 855 RFts were considered in our study.

### 2.5. Statistical Analysis and Model Building

The correlation between clinical variables, metabolic, and volumetric PET parameters and the outcome (i.e., FFR0 vs. FFR1) was investigated using Two-tailed Wilcoxon–Mann–Whitney U-type test (*p* < 0.05). All categorical variables correlation was investigated using Chi square test (*p* < 0.05).

Inter-scanner variability was investigated. Among 50 patients (42 FFR0, 8 FFR1), only FFR0 database belongs to two different scanners (34 scanned with scanner A, 8 with scanner B).

Before RFts reduction, the Synthetic Minority Over-Sampling Technique (SMOTE) strategy was used to balance the sample distribution of FFR0 and FFR1 into the dataset, obtaining a sample size of 84 lesions, 42 labeled FFR0, and 42 FFR1, as previously described [25]. The cohort was then randomly divided into a training set (70%) and an internal validation set (30%). The training set was used to build a model for the prediction of FFR response before surgery.

A filter feature selection algorithm using a hand-crafted python script was implemented to identify robust and non-redundant RFTs. The Bonferroni correction for multiple tests was used considering a *p*-value threshold < 0.001. Prior to the feature selection step, no data transformation or normalization was conducted. To evaluate the most robust, non-redundant and most reproducible RFts a *p* < Bonferroni threshold of the Wilcoxon–Mann–Whitney U type test was considered. In addition, the Spearman Rank correlation coefficient and the non-corrected *p*-Value were, respectively, calculated for completeness. This analysis was independently performed for both CT-based and PET-based RFts datasets. To assess the reliability and reproducibility of the selected RFts, their mean values were plotted, and correlation coefficient heatmaps were generated for the two different scanners. All the statistical analyses reported in the manuscript were performed using custom Python scripts (version 3.8) leveraging standard and widely used libraries such as pandas and scikit-learn.

The 10-fold cross-validation was used for the training of two independent ML Models. Two ML prediction models (PET Model, CT Model) were independently built. PET and CT Models were built using RFts selected and extracted from the corresponding images dataset (all VOIs for the PET Model and CT Model).

Orange data mining was used for the training, testing, and validation of three different ML Models using PET, CT, and clinical signatures, respectively. For each ML Model, three different algorithms were trained: Random Forest (RF), Support Vector Machine (SVM), and Decision Tree (Tree).

Area under the ROC curve (AUC), Classification Accuracy (CA), Precision (PREC), Recall (REC), and Specificity (SPEC) were calculated in the training and validation steps. In addition, Sensitivity (SEN), True-Positive (TP), and True-Negative (TN) were reported in validation. These performance scores were used to evaluate the best algorithm for CT and PET ML Model, respectively.

## 3. Results

### 3.1. Patients’ Characteristics

There were 25 men and 25 women with a mean age of 63.3 ± 14.2 years (SD). Most patients (29/50) underwent open surgery with sternotomy, 13 video-assisted thoracic surgery, and 8 robotic-assisted thoracic surgery. Furthermore, 14 out of 50 patients (28%) had a R1 resection, while 35 patients (70%) underwent radiotherapy of mediastinum region after surgery.

Based on WHO-classes of risk, we had 27 (54.0%) and 23 (46.0%) patients with LRT and HRT, respectively. This latter included also three patients with thymic carcinoma.

Masaoka system stage was available in 49 patients. According to this, 11 patients (22.4%) were classified with advanced disease (stage III or IV). Nineteen patients (38%) were associated also with diagnosis of myasthenia gravis. Moreover, median FFR according to Masaoka classification was 86, 34, 19, 13, and 33 months for stage I, IIa, IIb, III, and IV, respectively.

The median time interval between PET/CT and surgery was 60 days (range 29–83 days). Follow-up data from surgery to the onset of recurrence was collected for 50 patients.

Fifty patients with thymoma met the inclusion criteria. Overall, 50 thymic lesions detected by [^18^F]FDG PET/CT were considered for the analysis. Among them, 8 (16%) patients had recurrence (i.e., FFR1), while 42 did not (i.e., FFR0). Results are shown by mean and standard deviation for the two groups. Detailed characteristics of patients included are reported in Table 1.

### 3.2. [^18^F]FDG PET/CT Analysis

The results of the analysis of the parameters extracted from the PET of each patient to discriminate FFR0 vs. FFR1 are detailed in Table 2. Metabolic and volumetric parameters, such as SUVmax, SUVmean, SUVpeak, TLG, and MTV of the primary tumor were non-significant for discriminating clinical outcome, whereas only three variables were significant: rPET, qPET, and T/M ratio.

### 3.3. Radiomic Analysis and Model Building

Among the 855 RFts extracted from the original and filtered images, 43 robust RFts in the CT dataset and 16 in the PET dataset exhibited statistically significant differences between the non-FFR and FFR groups.

In both CT and PET datasets, the selected robust RFts predominantly belong to textural wavelet-based classes. The complete list of selected RFts is provided in the Supplementary Material (Tables S1 and S2). The high reproducibility and robustness of the selected features, independent of the acquisition scanner, are demonstrated in the Supplementary Material (Figures S1–S6) for CT and PET, respectively. The chosen hyperparameters for each learner are essentially listed in Supplementary Material (Text S1).

In 10-fold cross-validation, the results, averaged over the folds, are listed in terms of AUC, CA, precision, recall and specificity. The performances in all of the three learners showed AUC > 0.85 and CA > 0.83 in the CT Model, while an AUC > 0.89 and CA > 0.88 for the PET Model. In the training phase, the model that showed the better performance was the PET Model, considering RF classifier reach with AUC > 0.99 and CA > 0.94. Table 3 displays the performances of the three learners in 10-fold cross-validation across the two different models.

**Figure 2:**
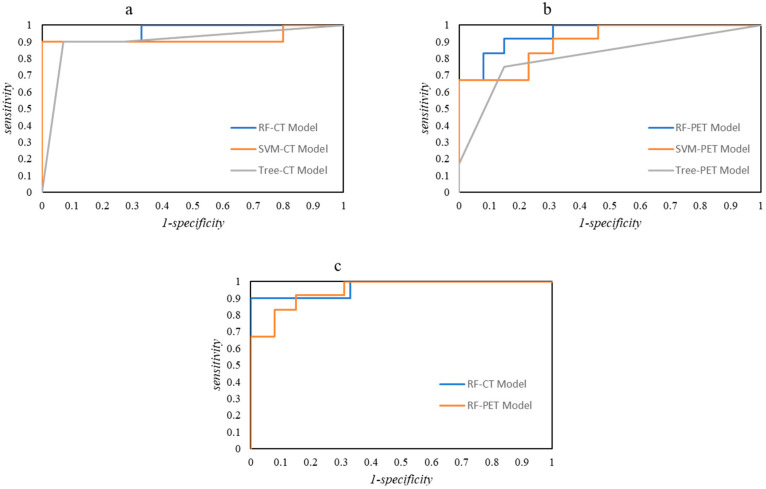
ROC curves of the three learners relative to the CT Model (**a**), PET Model (**b**), In (**c**), a comparison of ROC curves for the best learner (RF).

The classification performances in the validation step are summarized in Table 4. Overall, CT Models showed better results (AUC, CA, precision, sensitivity, specificity, TP, and TN all above 0.61) using all three algorithms. The best performances were obtained by RF into the CT Model (AUC = 0.97, CA = 0.88, Prec = 0.88, Recall = 0.88, Spec = 0.89, TN = 81.8%, TP = 92.9%). Also, the PET Model showed great performances. Even in this case the best classifier was RF and obtained AUC = 0.95, CA = 0.84, Prec = 0.84, Recall = 0.84, Spec = 0.84, TN = 83.3%, TP = 84.6%. ROC curves of the two ML Models are graphed in Figure 2.

Finally, a radial plot was built to make a visual comparison of the performance scores of the learners (i.e., RF, SVM, and Tree) for each of the two ML models trained (Figure S6). In addition, a shapely additive explanation (SHAP) analysis was graphed to demonstrate how features within the RF FFR classifier impacted the model output, in each of the two models (Figure S5).

**Figure 3:**
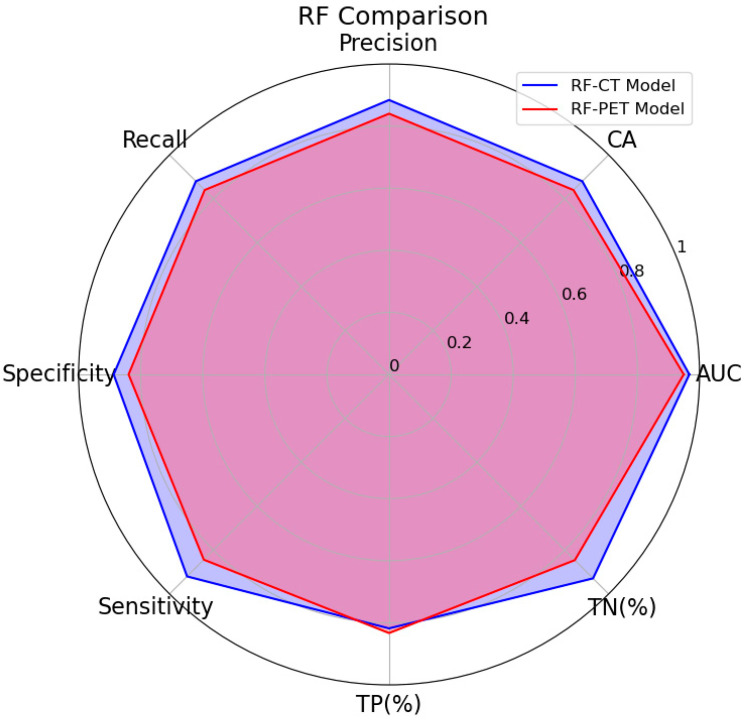
Radial plot of RF learner performances scores of ML Models.

Figure 3 represents a graphical comparison of the performances of RF, that resulted in the best learner in terms of performance between the two models.

## 4. Discussion

Although TETs are relatively uncommon, they represent the most frequently occurring primary neoplasm in the adult anterior mediastinum. The wide variation in morphology and oncologic behavior among TETs leads to accurate prognostic assessment, which is essential for guiding treatment decisions, particularly before surgery [3,26].

In recent decades, in the field of molecular imaging, several studies have been published on the potential role of ML approaches or DL models to predict histology, therapy assessment, or clinical outcomes in different malignancies [27,28,29,30].

In our study, we aimed to design a ML model able to evaluate prognosis in patients with TETs. To our best knowledge, this is the first study which has explored the potential role of ML models to preoperatively predict survival, expressed as FFR. As first study with this aim, comparison to other reports is difficult. Previously, Nakajo et al. [31] examined whether a ML approach using [^18^F]FDG PET-based radiomic and DL features could predict the pathological risk subtypes of TETs. In order to predict thymic carcinoma, accuracy was significantly higher in the logistic regression model compared to SUVmax, MTV and TLG, as well as the RF model was superior to MTV and TLG for predicting high-risk TETs. More recently, Gao et al. [22] developed a nomogram, based on the combination of clinical–radiological and radiomics features from CT images, to differentiate high-risk from low-risk thymomas in 178 patients before surgery. The nomogram model, which integrates clinico-radiological features (i.e., incomplete capsula) and seven radiomics signatures, demonstrated better diagnostic efficacy than any single model, with the respective AUC, accuracy, sensitivity, and specificity of 0.974, 0.921, 0.962, and 0.900 in the training cohort, and 0.960, 0.892, 0923, and 0.897 in the validation cohort, respectively.

In our study, a high number of robust RFts, able to discriminate between FFR0 and FFR1, were extracted from PET (i.e., n = 16) and CT images (i.e., n = 43). This finding highlights the added value of radiomic analysis in comparison to standard parameters currently applied in the daily clinical practice of thymomas imaging. Interestingly, all the robust RFts extracted from the PET dataset belong to the textural wavelet-based class (i.e., calculated for the filtered image/VOI). This finding reflects the fact that high- and low-frequency information extracted from PET images provides powerful informative data for use in a predictive model.

Robust radiomics signatures, extracted from PET and CT images, were used for training three ML models for each dataset. We applied 10-fold CV to increase the amount of data available for training the models. Interestingly, the RF Model was very accurate regarding the discrimination of the two groups, achieving very high performances, slightly superior for CT (AUC = 0.970; CA = 0.880; PRE = 0.884, REC = 0.880, SPEC = 0.887, SEN = 0.920, TP = 81.8%, TN = 92.9%) than PET models (AUC = 0.949; CA = 0.840; PRE = 0.842, REC = 0.846, SPEC = 0.839, SEN = 0.844, TP = 83.3%, TN = 84.6). Our results, if confirmed in external datasets, could potentially be useful in clinical practice for developing individualized therapeutic schedules.

Furthermore, in the present study, three clinico-metabolic variables (i.e., rPET, qPET, and T/M ratio) were significantly associated with FFR and incorporated in our model. These findings are consistent with our previous study, where the same parameters were predictors for FFR along with SUVmax and SUVmean [23]. In this regard, considering that in clinical practice traditional metabolic parameters still play a significant role for predicting clinical outcomes, our results may be useful to individuate patients who may really benefit from adjuvant therapy.

There are still some limitations in our research. Firstly, this study was a retrospective analysis, with selection bias being inevitable. Secondly, all the data were derived from a single center, and multi-center collaboration is desirable to collect larger samples and to improve diagnostic efficacy in the near future. Thirdly, sample imbalance exists; although SMOTE was employed to synthetically augment the minority class and mitigate this issue, such techniques can introduce artificial patterns that do not fully capture the complexity of real data. This may lead to model overfitting and reduced generalizability, particularly when applied to external datasets. Therefore, future studies should consider collecting more balanced datasets or adopting advanced resampling and cost-sensitive learning techniques to better handle class imbalance in a more realistic manner. Fourthly, diagnostic performance may be overestimated in the training cohort due to a lack of external validation. Regarding this aspect, although the availability of the data from two different scanners might seem a limitation, it is actually a strength of the study. In fact, the development of ML models trained on robust features on different scanners could improve the generalization of the model during the external validation step as demonstrated previously [32]. In this work, the hyperparameter optimization was performed following an empirical rule, based on all performances scores; future research will include the grid search technique in 10-fold cross-validation, to better optimize the automation process in a clinical prospective works [33].

## 5. Conclusions

To summarize, our study highlights that radiomic analysis and ML models achieved an optimal accuracy for predicting survival of patients with thymomas before surgery. The SHAP analysis conducted in this study, as recently demonstrated by Urso and colleagues [34], is essential to qualitatively visualize the impact of the features in the development of RF Model. As a non-invasive method, our model could serve as an effective tool to assist clinicians for treatment decisions. However, further studies should be made to validate our preliminary findings.

## Figures and Tables

**Table 1:** Characteristics of the study population.

Variables	FFR 0	FFR 1	Tot	*p*
	42 (84%)	8 (16%)	50	
Age	63.95 ± 12.27	56.25 ± 11.97	62.72 ± 12.43	
**Sex**				0.44
Male	20 (48%)	5 (63%)	25 (50%)	
Female	22 (52%)	3 (38%)	25 (50%)	
**FFR months**	41.36 ± 32.92	24 ± 15.6	38.88 ± 31.52	0.55
**WHO**				0.007
LR	27 (64%)	1 (13%)	28 (56%)	
HR	15 (36%)	7 (88%)	22 (44%)	
**Miastenia G**				0.105
No	24	7	31	
Yes	18	1	19	
**Masaoka**				<0.001
I	5	0	5	
IIa	25	0	25	
IIb	6	2	8	
III	5	2	7	
IV	1	4	5	
**8th TNM stage**				<0.001
I	36	2	38	
III	5	2	7	
IV	1	4	5	

FFR = free-from recurrence; LR = low-risk; HR = high risk.

**Table 2:** Relationship between metabolic parameters and clinical outcome.

	FFR 0	FFR 1	Tot	*p* ^1^
	42	8	50	
SUVmax	4.95 ± 1.94	8.39 ± 5.1	5.5 ± 2.91	>0.05
SUVmean	2.96 ± 1.02	4.92 ± 3.22	3.27 ± 1.7	>0.05
SUVpeak	4.14 ± 1.67	7.01 ± 3.97	4.6 ± 2.39	>0.05
TLG	207.36 ± 401.63	1829.02 ± 2986.12	466.83 ± 1330.22	>0.05
MTV	55.72 ± 84.13	376.36 ± 533.48	107.02 ± 246.33	>0.05
T_cm	4.92 ± 2.61	10.85 ± 7.89	5.87 ± 4.41	>0.05
rPET (T/L)	1.74 ± 0.72	3.62 ± 2.44	2.04 ± 1.33	**<0.001**
qPET (N/U)	1.79 ± 0.75	3.68 ± 2.11	2.09 ± 1.26	**<0.001**
SUVmax_T/Tcm	1.15 ± 0.53	1.74 ± 2.81	1.25 ± 1.18	>0.05
T/M	2.75 ± 1.12	5.86 ± 4.59	3.25 ± 2.32	**<0.001**

^1^: Bonferroni threshold = 5.74 × 10^−5^; FFR = free-from recurrence. Bold is to highlight parameters with statistical significance (i.e., < 0.05).

**Table 3:** Performances scores in 10-Fold CV for each ML Model.

	AUC	CA	PREC	REC	SPEC
**CT Model**					
RF	0.96	0.90	0.90	0.90	0.90
SVM	0.88	0.83	0.84	0.83	0.84
Tree	0.86	0.85	0.85	0.85	0.85
**PET Model**					
RF	0.99	0.95	0.95	0.95	0.95
SVM	0.98	0.88	0.88	0.88	0.88
Tree	0.90	0.92	0.92	0.92	0.92

AUC = area under the curve; CA = classification accuracy; PREC = precision; REC = recall; SPEC = specificity.

**Table 4:** Performances scores in the validation step for each ML Model.

	AUC	CA	PREC	REC	SPEC	SEN	TP (%)	TN (%)
**CT Model**								
RF	0.97	0.88	0.88	0.88	0.89	0.92	81.8	92.9
SVM	0.92	0.84	0.85	0.84	0.86	0.91	75.1	92.3
Tree	0.91	0.80	0.83	0.80	0.83	0.89	69.2	91.7
**PET Model**								
RF	0.95	0.84	0.84	0.85	0.84	0.84	83.3	84.6
SVM	0.90	0.77	0.76	0.76	0.76	0.77	75.0	76.9
Tree	0.81	0.80	0.80	0.80	0.80	0.79	81.8	78.6

AUC = area under the curve; CA = classification accuracy, PREC = precision, REC = recall, SEN = sensitivity, SPEC = specificity, TN = true negative; TP = true positive.

## Data Availability

No new data were created.

## Supplementary Materials

The following supporting information can be downloaded at: https://www.mdpi.com/article/10.3390/bioengineering12070721/s1. Text S1: Supplementary Methods: Model Hyperparameters. Table S1: Robust CT derived radiomic features. Table S2: Robust PET derived radiomic features. Figure S1: Inter scanner variability, mean CT robust RFts in Log scale. Figure S2: Heat-map, CT robust RFts correlation coefficient in Department A (left) and B (right). Figure S3: Inter scanner variability, mean PET robust RFts in Log scale. Figure S4: Heat-map, PET robust RFts correlation coefficient in Department A (left) and B (right). Figure S5: A radial plot to visually compare the performance scores of the three learners (i.e., RF, SVM and Tree) for each of the two ML trained model. Figure S6: SHAP features relevance in the RF of the two ML models. The graphed features are the top 10 relevant features in the RF-CT Model (a) and RF-PET Model (b). Features are ranked in order of their importance. Each dot represents a single patient’s prediction. Colors indicate the magnitude of the feature value in each row for a given patient. The x-axis indicates the model’s tendency for the prediction of FFR or non-FFR due to a given feature value in a given patient.
