# Supplementary material for: Machine Learning Models Derived from [18F]FDG PET/CT for the Prediction of Recurrence in Patients with Thymomas
Source: Bioengineering (Basel). 2025 Jun 30;12(7):721. doi: 10.3390/bioengineering12070721 (PMC12292471; doi:10.3390/bioengineering12070721)

Text S1: Supplementary Methods: Model Hyperparameters

The Random Forest model consisted of 100 trees with unrestricted feature consideration per split and a maximum tree depth of 3 to mitigate overfitting, while node splitting was not constrained by instance count. The Support Vector Machine employed a sigmoid kernel defined as tanh(auto x·y + 0.5), with a numerical tolerance of 0.02 for convergence and a maximum iteration limit of 100. The Decision Tree model applied pruning by requiring a minimum of 5 instances in internal nodes, limited tree depth to 100, and ceased splitting when the dominant class within a node exceeded 95%.

Table S1: Robust CT derived radiomic features.

Filter	Class	Feature	FFR 0	FFR 1	Tot	p-value	r
wavelet-LHH	glcm	InverseVariance	5.1E-1±4.87E-3	4.97E-1±1.11E-2	5.03E-1±1.08E-2	1.49E-05	0.174
wavelet-HLH	firstorder	10Percentile	-1.7E+1±3.56E+0	-2.09E+1±2.06E+0	-1.89E+1±3.51E+0	4.28E-05	-0.226
wavelet-HLH	firstorder	Entropy	1.46E+0±2.67E-1	1.72E+0±1.35E-1	1.59E+0±2.46E-1	5.2E-05	-0.054
wavelet-HLH	firstorder	MeanAbsoluteDeviation	1.15E+1±3.29E+0	1.43E+1±1.53E+0	1.29E+1±2.91E+0	2.35E-05	-0.055
wavelet-HLH	firstorder	Minimum	-9.48E+1±4.78E+1	-1.8E+2±5.35E+1	-1.38E+2±6.63E+1	4.93E-07	-0.07
wavelet-HLH	firstorder	Range	2.07E+2±8.43E+1	3.67E+2±1.18E+2	2.87E+2±1.3E+2	1.27E-06	-0.07
wavelet-HLH	firstorder	RootMeanSquared	1.58E+1±5.13E+0	2.08E+1±2.97E+0	1.83E+1±4.87E+0	1.57E-05	0.004
wavelet-HLH	firstorder	Variance	2.75E+2±1.94E+2	4.48E+2±1.26E+2	3.62E+2±1.85E+2	1.09E-05	0.023
wavelet-HLH	glcm	Autocorrelation	2.58E+1±2.2E+1	7.57E+1±3.97E+1	5.07E+1±4.06E+1	1.04E-07	-0.069
wavelet-HLH	glcm	ClusterProminence	9.83E+0±1.5E+1	2.99E+1±2.2E+1	1.99E+1±2.13E+1	3.18E-05	0.028
wavelet-HLH	glcm	ClusterTendency	1.04E+0±6.08E-1	1.51E+0±4.07E-1	1.27E+0±5.66E-1	2.35E-05	-0.028
wavelet-HLH	glcm	Contrast	1.01E+0±5.42E-1	1.47E+0±3.83E-1	1.24E+0±5.21E-1	1.28E-05	0.04
wavelet-HLH	glcm	DifferenceAverage	6.73E-1±1.4E-1	7.99E-1±7.83E-2	7.36E-1±1.29E-1	1.64E-05	-0.016
wavelet-HLH	glcm	DifferenceEntropy	1.34E+0±2.2E-1	1.55E+0±1.2E-1	1.45E+0±2.05E-1	4.08E-05	-0.049
wavelet-HLH	glcm	DifferenceVariance	5.18E-1±3.1E-1	8.03E-1±2.59E-1	6.61E-1±3.18E-1	2.74E-05	0.054
wavelet-HLH	glcm	Id	7.03E-1±3.07E-2	6.73E-1±1.58E-2	6.88E-1±2.87E-2	2.88E-05	-0.142
wavelet-HLH	glcm	Idm	6.93E-1±3.84E-2	6.55E-1±1.97E-2	6.74E-1±3.57E-2	3.52E-05	-0.144
wavelet-HLH	glcm	JointAverage	4.7E+0±1.93E+0	8.3E+0±2.19E+0	6.5E+0±2.74E+0	2.09E-07	-0.114
wavelet-HLH	glcm	JointEntropy	2.78E+0±4.62E-1	3.25E+0±2.61E-1	3.01E+0±4.42E-1	4.73E-05	-0.065
wavelet-HLH	glcm	SumAverage	9.41E+0±3.85E+0	1.66E+1±4.39E+0	1.3E+1±5.47E+0	2.48E-07	-0.085

wavelet-HLH	glcm	SumSquares	4.73E-1±2.42E-1	7.03E-1±2.03E-1	5.88E-1±2.5E-1	1.64E-05	0.022
wavelet-HLH	gldm	GrayLevelVariance	5.43E-1±3.04E-1	8.08E-1±2.01E-1	6.75E-1±2.89E-1	1.15E-05	0.02
wavelet-HLH	gldm	HighGrayLevelEmphasis	2.63E+1±2.2E+1	7.64E+1±3.98E+1	5.14E+1±4.07E+1	9.27E-08	-0.073
wavelet-HLH	gldm	LargeDependenceHighGrayLevelEmphasis	3.22E+3±2.93E+3	8.56E+3±4.6E+3	5.89E+3±4.68E+3	2.9E-06	-0.256
wavelet-HLH	gldm	LargeDependenceLowGrayLevelEmphasis	1.0E+1±8.66E+0	2.33E+0±1.56E+0	6.17E+0±7.29E+0	5.51E-07	0.005
wavelet-HLH	gldm	LowGrayLevelEmphasis	7.95E-2±6.03E-2	2.36E-2±2.03E-2	5.15E-2±5.28E-2	1.15E-05	-0.162
wavelet-HLH	gldm	SmallDependenceHighGrayLevelEmphasis	1.25E+0±1.14E+0	4.31E+0±2.51E+0	2.78E+0±2.48E+0	6.9E-08	-0.006
wavelet-HLH	glrlm	GrayLevelVariance	6.59E-1±3.79E-1	1.03E+0±2.7E-1	8.46E-1±3.77E-1	1.09E-05	0.017
wavelet-HLH	glrlm	HighGrayLevelRunEmphasis	2.66E+1±2.21E+1	7.68E+1±3.99E+1	5.17E+1±4.08E+1	9.82E-08	-0.064
wavelet-HLH	glrlm	LongRunHighGrayLevelEmphasis	9.85E+1±8.67E+1	2.56E+2±1.31E+2	1.77E+2±1.36E+2	2.9E-06	-0.193
wavelet-HLH	glrlm	LongRunLowGrayLevelEmphasis	3.13E-1±2.76E-1	7.31E-2±5.17E-2	1.93E-1±2.31E-1	5.83E-07	-0.05
wavelet-HLH	glrlm	LowGrayLevelRunEmphasis	8.09E-2±6.14E-2	2.42E-2±2.12E-2	5.26E-2±5.38E-2	1.41E-05	-0.166
wavelet-HLH	glrlm	ShortRunHighGrayLevelEmphasis	1.9E+1±1.57E+1	5.66E+1±2.96E+1	3.78E+1±3.02E+1	3.8E-08	-0.078
wavelet-HLH	glrlm	ShortRunLowGrayLevelEmphasis	5.7E-2±4.25E-2	1.85E-2±1.71E-2	3.78E-2±3.76E-2	4.96E-05	-0.263
wavelet-HLH	glszm	HighGrayLevelZoneEmphasis	2.98E+1±2.22E+1	8.18E+1±4.18E+1	5.58E+1±4.23E+1	1.1E-07	-0.059
wavelet-HLH	glszm	LowGrayLevelZoneEmphasis	1.54E-1±1.49E-1	3.89E-2±3.74E-2	9.66E-2±1.23E-1	5.74E-05	-0.29
wavelet-HLH	glszm	SmallAreaHighGrayLevelEmphasis	1.77E+1±1.33E+1	4.79E+1±2.6E+1	3.28E+1±2.55E+1	4.16E-07	-0.097
wavelet-HLH	glszm	SmallAreaLowGrayLevelEmphasis	1.02E-1±1.05E-1	2.43E-2±2.26E-2	6.32E-2±8.49E-2	5.74E-05	-0.28
wavelet-HLH	ngtdm	Complexity	2.72E+1±2.02E+1	8.44E+1±5.65E+1	5.58E+1±5.1E+1	2.22E-07	-0.097
wavelet-HHH	glcm	Idn	8.94E-1±2.47E-2	9.18E-1±1.61E-2	9.06E-1±2.41E-2	4.73E-05	-0.123
wavelet-HHH	glrlm	ShortRunHighGrayLevelEmphasis	5.19E+0±2.46E+0	1.01E+1±5.35E+0	7.64E+0±4.82E+0	5.2E-05	-0.032
wavelet-HHH	glszm	ZoneEntropy	1.89E+0±4.28E-1	2.58E+0±5.17E-1	2.24E+0±5.83E-1	4.08E-05	0.165
wavelet-HHH	ngtdm	Complexity	4.42E+0±2.58E+0	1.11E+1±7.09E+0	7.76E+0±6.28E+0	6.48E-06	-0.172

Table S2: Robust PET derived radiomic features.

Filter	Class	Feature	FFR 0	FFR 1	Tot	p-value	r
wavelet-LLH	firstorder	90Percentile	3.34E+1±1.52E+2	5.73E-1±2.33E-1	1.7E+1±1.08E+2	4.65E-05	0.211
wavelet-LLH	firstorder	Variance	4.47E+4±2.05E+5	8.4E-1±9.43E-1	2.23E+4±1.46E+5	3.12E-05	0.218
wavelet-LHL	firstorder	90Percentile	3.76E+1±1.69E+2	1.08E+0±1.07E+0	1.93E+1±1.2E+2	2.97E-05	0.253
wavelet-LHL	firstorder	Energy	1.42E+7±6.81E+7	1.08E+3±1.12E+3	7.08E+6±4.84E+7	4.01E-05	-0.089
wavelet-LHL	firstorder	Maximum	8.34E+1±3.77E+2	1.96E+0±1.39E+0	4.27E+1±2.68E+2	4.01E-05	0.032
wavelet-LHL	firstorder	Minimum	-1.4E+2±6.3E+2	-3.24E+0±1.85E+0	-7.17E+1±4.48E+2	1.24E-05	0.039
wavelet-LHL	firstorder	Range	2.24E+2±1.01E+3	5.2E+0±3.21E+0	1.14E+2±7.16E+2	7.76E-06	0.079
wavelet-LHL	firstorder	TotalEnergy	9.57E+8±4.61E+9	7.27E+4±7.57E+4	4.79E+8±3.27E+9	3.69E-06	-0.099
wavelet-LHH	glszm	GrayLevelNonUniformityNormalized	4.88E-1±1.08E-1	5.54E-1±4.02E-2	5.21E-1±8.77E-2	1.8E-05	0.047
wavelet-LHH	glszm	GrayLevelVariance	1.72E+1±7.69E+1	2.23E-1±2.01E-2	8.73E+0±5.47E+1	1.8E-05	0.052
wavelet-LLL	glcm	ClusterShade	-2.02E+5±9.49E+5	8.05E-2±1.18E-1	-1.01E+5±6.75E+5	4.99E-07	0.038
wavelet-LLL	glcm	InverseVariance	5.86E-4±2.2E-3	6.65E-2±9.98E-2	3.36E-2±7.76E-2	1.42E-05	-0.071
wavelet-LLL	glcm	MCC	9.79E-1±1.36E-1	7.2E-1±2.72E-1	8.49E-1±2.51E-1	8.32E-06	0.04
wavelet-LLL	glszm	SizeZoneNonUniformityNormalized	9.86E-1±7.78E-2	7.45E-1±2.4E-1	8.65E-1±2.15E-1	6.35E-06	-0.031
wavelet-LLL	glszm	ZoneVariance	1.47E+4±9.5E+4	2.22E+6±6.72E+6	1.12E+6±4.85E+6	4.54E-06	0.066
wavelet-LLL	ngtdm	Busyness	4.5E-2±2.91E-1	4.01E+0±5.95E+0	2.03E+0±4.64E+0	1.79E-06	-0.086

Figure S1: Inter scanner variability, mean CT robust RFts in Log scale

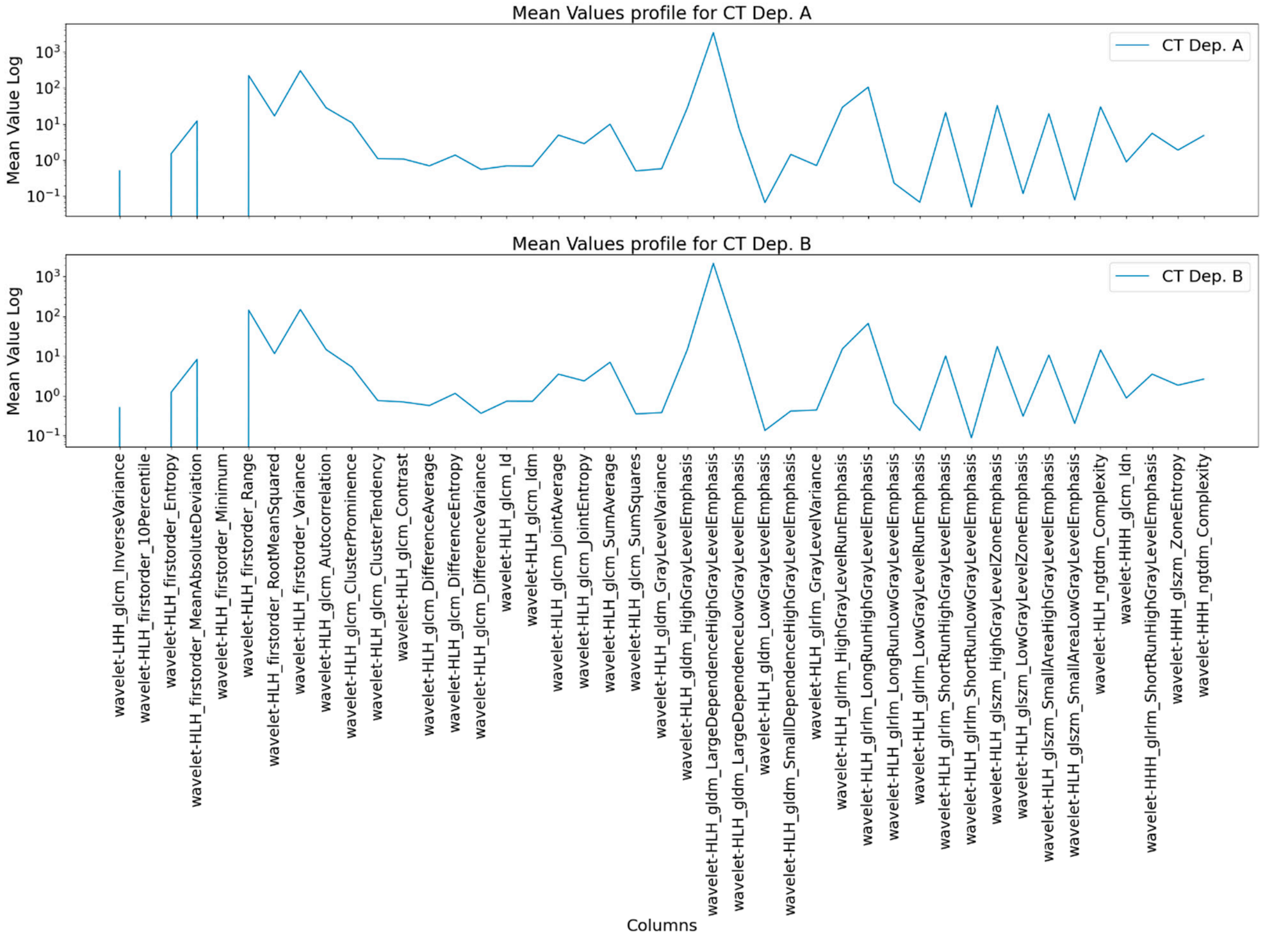

Figure S2: Heat-map, CT robust RFts correlation coefficient in Department A (left) and B (right)

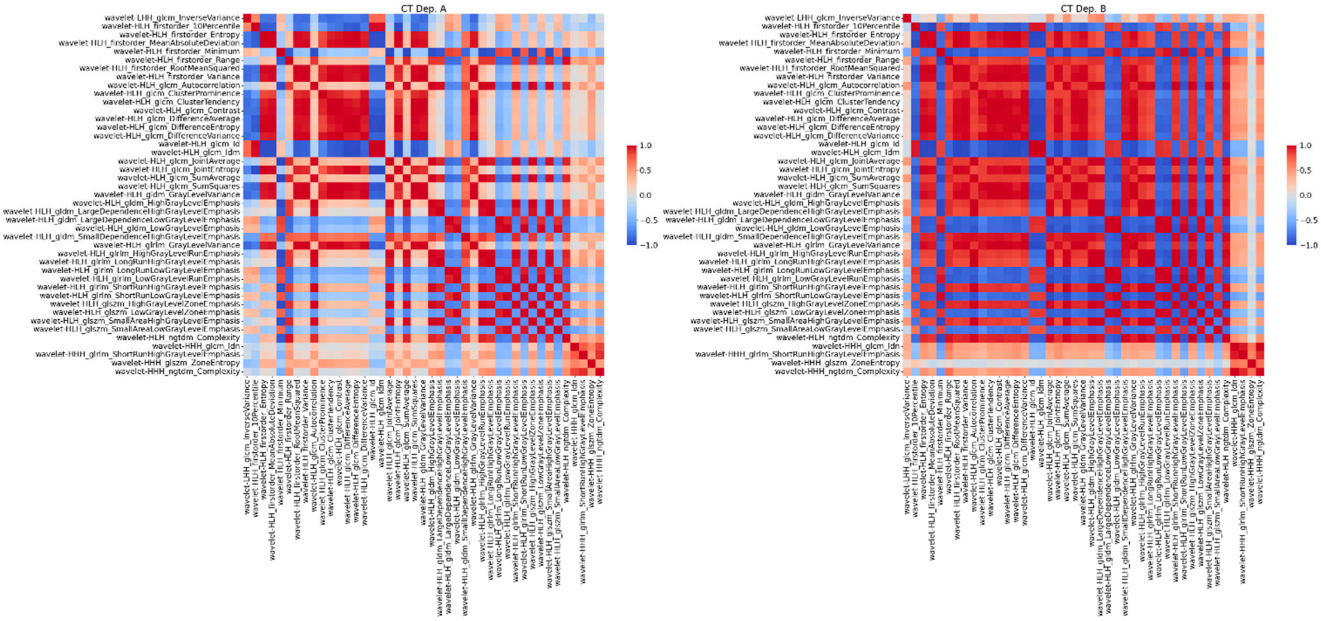

Figure S3: Inter scanner variability, mean PET robust RFts in Log scale

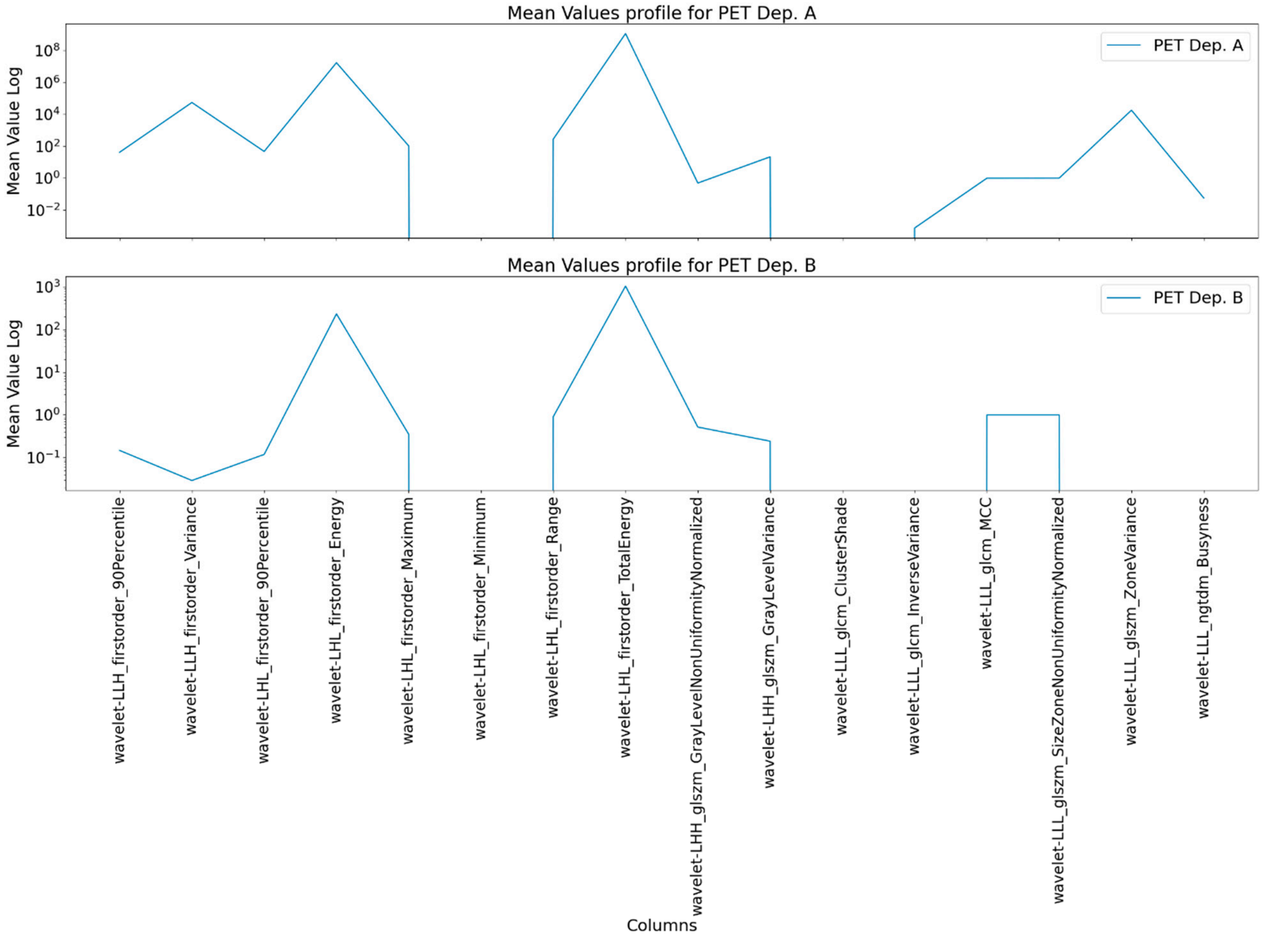

Figure S4: Heat-map, PET robust RFts correlation coefficient in Department A (left) and B (right)

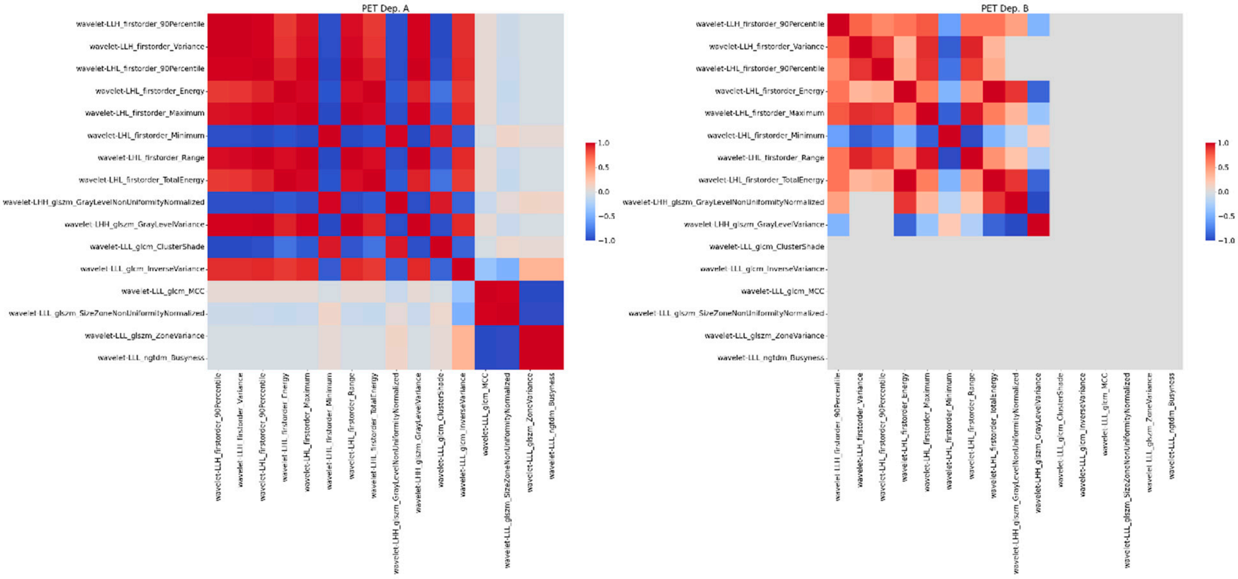

Figure S5. A radial plot to visually compare the performance scores of the three learners (i.e. RF, SVM and Tree) for each of the two ML trained model.

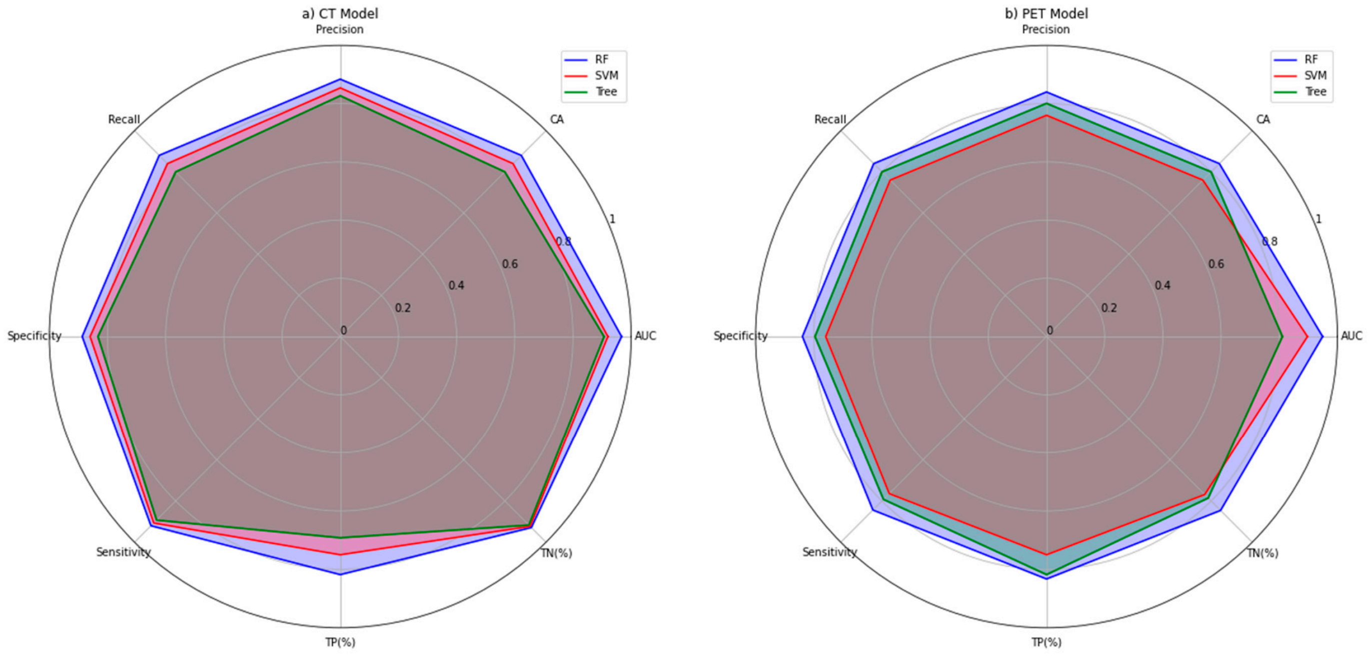

Figure S6. SHAP features relevance in the RF of the two ML models. The graphed features are the top 10 relevant features in the RF-CT Model (a) and RF-PET Model (b). Features are ranked in order of their importance. Each dot represents a single patient's prediction. Colors indicate the magnitude of the feature value in each row for a given patient. The x-axis indicates the model's tendency for the prediction of FFR or non-FFR due to a given feature value in a given patient.

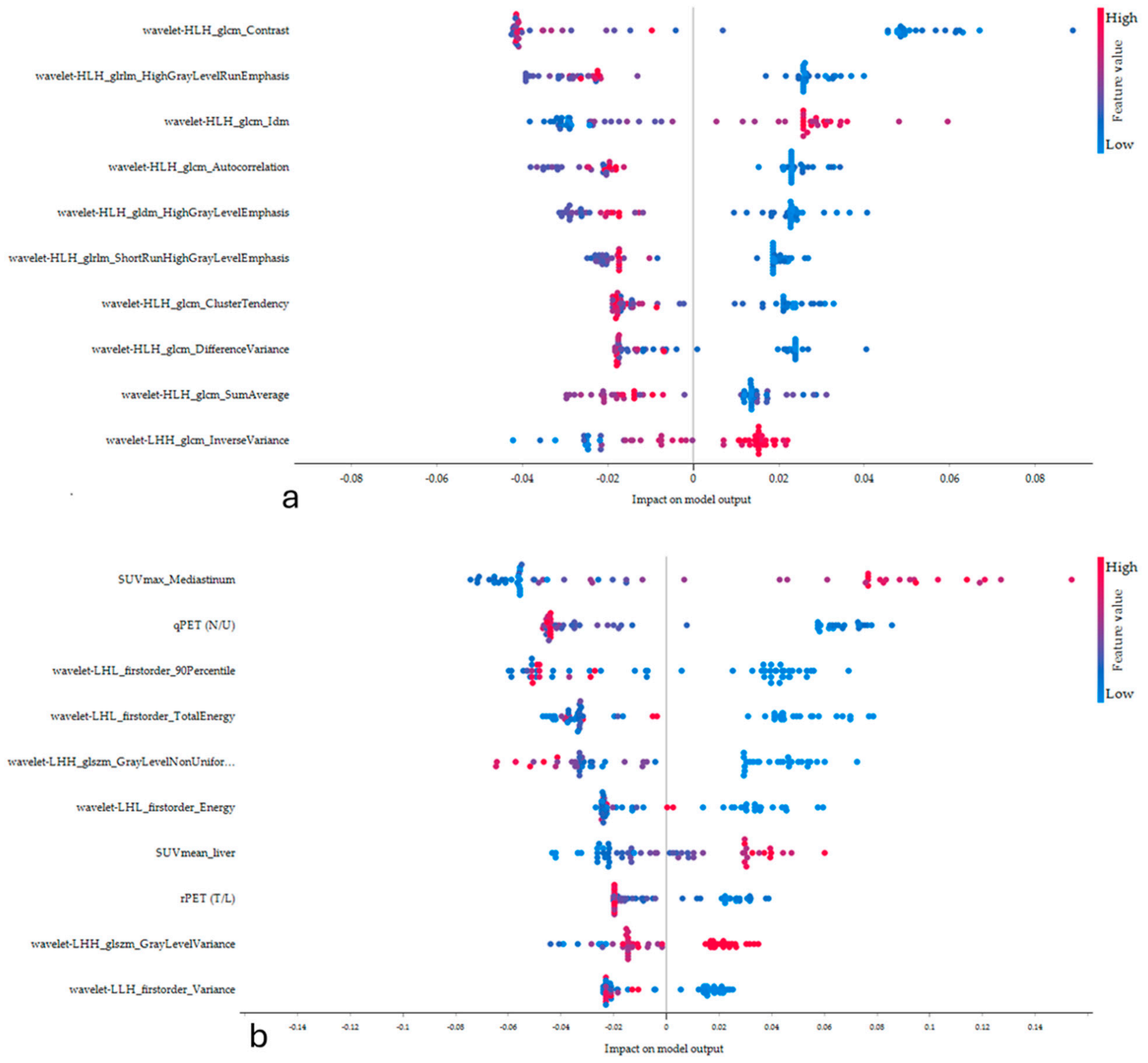

Supplement: Supplementary file 1 [file bioengineering-12-00721-s001.zip › bioengineering-3683134-supplementary.pdf]
